# A Fuzzy Adaptive Tightly-Coupled Integration Method for Mobile Target Localization Using SINS/WSN

**DOI:** 10.3390/mi7110197

**Published:** 2016-11-02

**Authors:** Wei Li, Hai Yang, Mengbao Fan, Chengming Luo, Jinyao Zhang, Zhuoyin Si

**Affiliations:** 1 School of Mechatronic Engineering, China University of Mining and Technology, Xuzhou 221116, Jiangsu, China; weilicmee@cumt.edu.cn (W.L.); wuzhi3495@cumt.edu.cn (M.F.); zhangjycumt@126.com (J.Z.); sizhuoyin@cumt.edu.cn (Z.S.); 2 College of Internet of Things Engineering, Hohai University, Changzhou 213022, Jiangsu, China; luocm@hhu.edu.cn

**Keywords:** wireless sensor network (WSN), strapdown inertial navigation system (SINS), mobile target, integrated positioning, tightly-coupled integration, fuzzy adaptive, Kalman filter

## Abstract

In recent years, mobile target localization for enclosed environments has been a growing interest. In this paper, we have proposed a fuzzy adaptive tightly-coupled integration (FATCI) method for positioning and tracking applications using strapdown inertial navigation system (SINS) and wireless sensor network (WSN). The wireless signal outage and severe multipath propagation of WSN often influence the accuracy of measured distance and lead to difficulties with the WSN positioning. Note also that the SINS are known for their drifted error over time. Using as a base the well-known loosely-coupled integration method, we have built a tightly-coupled integrated positioning system for SINS/WSN based on the measured distances between anchor nodes and mobile node. The measured distance value of WSN is corrected with a least squares regression (LSR) algorithm, with the aim of decreasing the systematic error for measured distance. Additionally, the statistical covariance of measured distance value is used to adjust the observation covariance matrix of a Kalman filter using a fuzzy inference system (FIS), based on the statistical characteristics. Then the tightly-coupled integration model can adaptively adjust the confidence level for measurement according to the different measured accuracies of distance measurements. Hence the FATCI system is achieved using SINS/WSN. This innovative approach is verified in real scenarios. Experimental results show that the proposed positioning system has better accuracy and stability compared with the loosely-coupled and traditional tightly-coupled integration model for WSN short-term failure or normal conditions.

## 1. Introduction

In the past several years, mobile target localization in an enclosed environment has received a lot of attention in many fields, such as indoor pedestrian navigation [1], coal mine automation [2], mobile robot navigation, and other fields. Moreover, wireless sensor network (WSN) has enormous potential for short-range positioning in an enclosed environment based on intelligence, networking, and distribution. WSN is comprised of a mobile node and multiple anchor nodes through a self-organized multi-hop. Anchor nodes detect the wireless signal, which is transmitted by mobile node; however, the wireless signal is liable to be influenced by the barrier, floor, and ceiling through multipath wireless channels and non-line-of-sight (NLOS) [3]. Among them, the barrier causes the most serious influence. It can block the wireless signal and cause gross error or non-measurement for some anchor nodes. Gharghan et al. [4] proposed a measured distance model between the mobile node and anchor node and improved the distance estimation accuracy with log-normal shadowing algorithm. Typically, the WSN cannot calculate the accurate position based on the low-accuracy distance estimation. If the number of anchor nodes that have received the wireless signal from the mobile node is less than four on a two-dimensional plane, the WSN cannot solve a final position result using the time of arrival (TOA) model. 

In contrast to WSN, the strapdown inertial navigation system (SINS) can provide continuous positioning information by processing inertial measurement unit (IMU) measurements without any external aids after the required initialization and alignment [5]. The SINS can express the ability of independent positioning and is used in many fields such as military arms, aerospace, indoor mobile tracking [6], and so on. However, the positioning accuracy of SINS degrades rapidly over time due to integration with IMU measured errors, especially for low-cost IMUs [7]. Sensor errors of IMU can be easily parameterized, but can only be precisely estimated and compensated using external information, such as GPS observations for outdoors or WSN measurements for indoors [8]. Therefore, considerable research effort has been focused on the SINS/WSN integrated positioning system recently. Hur [9] has built the IMU/WSN localization model with discrete-time for a mobile robot. Correa [10] have developed an enhanced extended Kalman filter (EKF) method for indoor positioning H_∞_ filter applications using WSN and IMU. Yang [11] proposed a fuzzy adaptive Kalman filter positioning system based on INS and WSN integration to estimate the position of a mobile target indoors. However, for the above methods, the WSN and SINS work independently and the fusion model combines their positions and velocities, if the WSN finishes calculating the effective position. Otherwise, the integrated positioning system will result in failure by means of the positions of WSN and SINS, when the WSN does not calculate the accurate position. That integrated method has been defined as a loosely-coupled integrated method according to [12]. 

In order to overcome the poor observability of measurement information for loosely-coupled integration, Ascher [13] proposed a tightly-coupled UWB/INS system for pedestrian indoor applications. Xu [14] has built a tightly-coupled integrated model with a Kalman filter (KF) for the INS/WSN system. The above integrated positioning systems based on a tightly-coupled integration scheme utilize the differences between the distances from the mobile node to the anchor nodes measured by SINS and those measured by WSN. However, their positioning accuracies are highly dependent on the accuracy of the distances measured, and differences are used as the measurement information for KF. Note that the measurement errors of WSN can deteriorate the overall positioning accuracy. Additionally, the tightly-coupled integrated system can be improved by the provision of more accurate measured distances through correcting the residual correlated errors. Dwivedi [15] estimated clock errors and range between two nodes simultaneously. Miloccol [16] proposed an efficient pseudo-optimal low-power based distance estimation method for the measured distance error of WSN. Go [17] and Li [18] discussed the accuracy of wireless localization, which is influenced by NLOS errors in WSN. However, the above proposed methods involve very complicated calculations and cannot be used directly for the integrated positioning system.

As for the tightly-coupled integrated positioning model, the statistical covariance of observation noise can reflect the measured accuracy for the distance between anchor node and mobile node. Meanwhile, the accuracy of the measured distance is certainly one deciding factor for the performance of a tightly-coupled integration model. However, the estimated accuracy for the distance between the mobile node and anchor node will be changed based on the varied real distance. Every anchor node reads the distances with different estimated accuracies according to the varied actual distances from the mobile node to every anchor node. Aiming at different measured accuracies for WSN, this paper proposed a fuzzy adaptive tightly-coupled integration (FATCI) positioning system based on the distance statistical covariance matrix with a fuzzy adaptive Kalman filter (FAKF). The tightly-coupled integrated positioning model is built through analyzing the loosely-coupled integrated model with SINS/WSN. Moreover, the error-corrected model for measured distance is built according to the basic operating principles of WSN. The fuzzy adaptive control strategy is configured to take advantage of the statistical information of the measured distance. As a consequence, the measurement covariance matrix of Kalman filter is tuned with the accuracy of measured distance. In this case, the confidence coefficient for every measurement is adjusted based on the fuzzy inference system. Then the FATCI model with SINS/WSN systems would be achieved.

The rest of the paper is organized as follows. The problem statement for loosely-coupled integrated model is presented in Section 2. Section 3 describes a model-updated algorithm for measured distance error based on the least squares regression, while Section 4 represents a FATCI model using the SINS/WSN system. In Section 5, we examine the performance of the proposed method and compare it to loosely-coupled and traditional tightly-coupled models. Finally, Section 6 concludes the paper. 

## 2. Problem Statement

**Figure 1:**
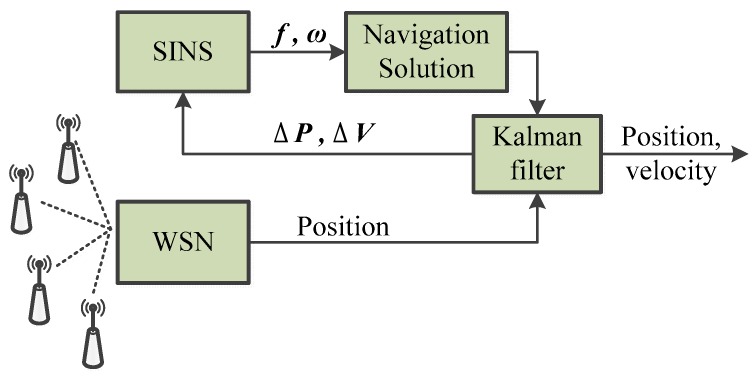
Structure of loosely-coupled integration model.

In this section, we focus on a SINS/WSN loosely-coupled integrated positioning system with the aim of analyzing its localization performance. It is clear that the loosely-coupled integrated method is a simple integration mode for the SINS/WSN integrated positioning system; its structure is shown as Figure 1. For the loosely-coupled method, SINS and WSN work independently. Firstly, the equations for error propagation of SINS are used for the state equation of the Kalman filter. Secondly, the measurement equation of the Kalman filter combines their measured data, which includes the positions and velocities of SINS and WSN, respectively. Finally, the optimal fusion result of the Kalman filter is utilized to correct the drifted error of SINS. The loosely-coupled mode operates with a simple rule and can be easily applied to engineering.

### 2.1. State Equation

According to the well-known Newton’s second law, the discrete time equations of motion for mobile target are illustrated as follows:

(1) $$\{\begin{array}{c} P_{k+1}^{n}=P_{k}^{n}+V_{k}^{n}T+C_{b}^{n}a_{k}^{b}T^{2}/2\\ V_{k+1}^{n}=V_{k}^{n}+C_{b}^{n}a_{k}^{b}T \end{array},$$

where $P^{n}$ and $V^{n}$ are the three-dimensional position and velocity of mobile target in the navigation coordinate (*n*-frame, *O_n_x_n_y_n_z_n_*) respectively; $a^{b}$ is the three-dimensional acceleration measured by SINS in the body coordinate (*b*-frame, *O_b_x_b_y_b_z_b_*); $C_{b}^{n}$ is the *b*-*n* frame transformation matrix; *T* is the sampling time of SINS; and *k* stands for the time index.

Equation (1) operates with the perfect differential for its parameters and the result is expressed as Equation (2):

(2) $$\{\begin{array}{c} \delta P_{k+1}^{n}=\delta P_{k}^{n}+\delta V_{k}^{n}T-\left(C_{b}^{n}a_{k}^{b}\times \right)\frac{T^{2}}{2}\delta A_{k}+C_{b}^{n}\frac{T^{2}}{2}\delta a_{k}^{b}\\ \;\delta V_{k+1}^{n}=\delta V_{k}^{n}-\left(C_{b}^{n}a_{k}^{b}\times \right)T\delta A_{k}+C_{b}^{n}T\delta a_{k}^{b} \end{array},$$

where $\delta P^{n}$ and $\delta V^{n}$ are the vectors of position error and velocity error in the *n*-frame, respectively; δA is the vector of attitude error, and the attitude angles are yaw (φ), pitch (θ), and roll (γ); $\delta a^{b}$ is a measured noise of accelerometer in the *b*-frame; and $a^{b}\times$ is expressed as an antisymmetric matrix for the acceleration vector.

The navigation filter is an error state space Kalman filter, closed loop filter, based on [19,20], where the inertial data is used to estimate position, velocity, and attitude. Additionally, let us consider the following state space representation of mobile target. First, a state variable is defined as:

(3) $$x_{k}={\left[\begin{array}{ccc} \delta P_{k}^{n} & \delta V_{k}^{n} & \delta A_{k} \end{array}\right]}^{T}.$$

So the state equation for the Kalman filter can be obtained as in Equation (4):

(4) $$x_{k}=F_{k,k+1}x_{k}+G_{k}W_{k},$$

where $W_{k}$ is the system noise of state equation, and its vector form is expressed as $W_{k}={\left[\begin{array}{cc} \omega_{\varepsilon } & \delta a^{b} \end{array}\right]}_{k}^{T}$; $\omega_{\varepsilon }$ is the bias of gyroscope which is a component of IMU; $G_{k}$ is a one-step transition disturbance matrix; and $F_{k,k+1}$ is a one-step transition matrix. Let us define them as:

(5) $$F_{k,k+1}=\left[\begin{array}{ccc} I_{3\times 3} & T\cdot I_{3\times 3} & -\left(C_{b}^{n}a_{k}^{b}\times \right)T^{2}/2\\ 0_{3\times 3} & I_{3\times 3} & -\left(C_{b}^{n}a_{k}^{b}\times \right)T\\ 0_{3\times 3} & 0_{3\times 3} & I_{3\times 3} \end{array}\right]$$

(6) $$G_{k}=\left[\begin{array}{cc} 0_{3\times 3} & C_{b}^{n}T^{2}/2\\ 0_{3\times 3} & C_{b}^{n}T\\ C_{b}^{n} & 0_{3\times 3} \end{array}\right].$$

### 2.2. Measurement Equation

The position of the mobile target can be measured by the WSN, which is estimated by Equation (7). In practice, the measurement vector ${\hat{P}}_{W,k}$ at sampling time *k* has the measured noise and the relationship is represented with the vector $V_{W,k}$ as follows:

(7) $${\hat{P}}_{W,k}=P_{W}+V_{W,k}$$

(8) $$V_{W,k}={\left[\begin{array}{ccc} \delta P_{W,x} & \delta P_{W,y} & \delta P_{W,z} \end{array}\right]}_{k}^{T}.$$

According to the IMU readings, the position of the mobile target can be calculated through the navigation solution algorithm, which is expressed as Equation (9). $V_{I,k}$ is the calculated error at sampling time *k*. Namely,

(9) $${\hat{P}}_{I,k}=P_{I}+V_{I,k}$$

(10) $$V_{I,k}={\left[\begin{array}{ccc} \delta P_{I,x} & \delta P_{I,y} & \delta P_{I,z} \end{array}\right]}_{k}^{T}.$$

For the loosely-coupled integrated model, the measurement of the SINS/WSN system is the set of difference between the positions from WSN and SINS. The measurement equation can be obtained based on Equations (7) and (9), expressed as:

(11) $$z_{k}=H_{k}x_{k}+v_{k},$$

where $z_{k}={\hat{P}}_{I,k}-{\hat{P}}_{W,k}$, $v_{k}$ is the measured noise and $v_{k}=V_{I,k}-V_{W,k}$. The observation matrix $H_{k}$ is denoted as

(12) $$H_{k}=\left[\begin{array}{cc} I_{3\times 3} & 0_{3\times 6} \end{array}\right].$$

Note that the easiest integration technique for position measurements, as known from GPS/INS integration algorithms, is the loosely-coupled approach. Here measurements first go through the position calculation (Figure 1) and the result is combined with the inertial sensor data in an error state Kalman filter. This model works only if more than three range measurements are available on a two-dimensional plane. This is why we propose the use of a tightly-coupled approach instead.

## 3. Measurement Model of WSN

In this section, we discuss a TOA-based measured distance error model of WSN to be taken into account the statistics of the measurement. In addition, according to the error model, an error corrected algorithm for the systematic error of measured distance is applied based on the least squares regression (LSR). Finally, we design some experiments to evaluate the performance for the measured distance model and its error-corrected algorithm.

### 3.1. Measured Distance Model of WSN

In a three-dimensional field, *N* anchor nodes with known locations are deployed around the to-be-located node, which is called a mobile node. In a localization process, the position and measured distance can be obtained, where ${\tilde{d}}_{ij}$ represents the measured distance between *i*-th anchor node and *j*-th mobile node, and can be derived by the TOA method. The position of the *i*-th anchor node is denoted as (*x_i_*, *y_i_*, *z_i_*). Let $d_{ij}$ be the true distance, then the relationship between ${\tilde{d}}_{ij}$ and $d_{ij}$ is

(13) $${\tilde{d}}_{ij}=fd_{ij}+\varepsilon_{ij}+c_{ij}+\Delta_{ij},$$

where *f* is the scale factor from real value to measured distance; $\varepsilon_{ij}$ is the potential NLOS error. If the propagation complies with line-of-sight (LOS) propagation, $\varepsilon_{ij}$ is zero; $c_{ij}$ is the system bias of WSN, which is always a constant value; and $\Delta_{ij}\sim N\left(0,\sigma_{ij}^{2}\right)$ is the additive white Gaussian noise with variance $\sigma_{ij}$.

### 3.2. Model Updating for Measured Distance Error

According to the TOA-based measured distance model of WSN, the potential NLOS error can be removed for the LOS propagation condition. Meanwhile the additive white Gaussian noise $\Delta_{ij}$ can be decreased by a Kalman filter. Hence the system bias $c_{ij}$ and the scale factor *f* have not been compensated for based on any filter algorithm. However, both the system bias $c_{ij}$ and the scale factor *f* are not changed with measuring times and location scene; they will be influenced by the wireless sensors. According to the above characteristics, the LSR algorithm can be used to correct the system bias $c_{ij}$ and the scale factor *f* with a large number of measurements.

From Equation (13), we can assume that

(14) $${\tilde{d}}_{i}=fd_{i}+c_{i}+\Delta_{i}\;\mathrm{for}\;i=1,2,...,N,$$

where the collection $\left\{\Delta_{i}\right\}$ is a random sample from a distribution with mean zero and standard deviation $\sigma_{i}$, and the other parameters (e.g., $f,c_{i},\;\mathrm{and}\;\sigma_{i}$) are unknown.

Least squares (LS) is a general estimation method introduced by Legendre in the early 1800s [21]. In the simple linear case, the LS estimators of *f* and *c_i_* are obtained by means of minimizing the following sum of squared deviations of observed from expected responses:

(15) $$S\left(f,c\right)=\sum_{i=1}^{N}{\left({\tilde{d}}_{i}-\left(fd_{i}+c_{i}\right)\right)}^{2}.$$

The goal is to estimate values of *f* and *c_i_* that minimize the error. In multivariable calculus we learn that this requires us to find the values of (*f*, *c_i_*) such that

(16) $$\frac{\partial S}{\partial f}=0,\;\frac{\partial S}{\partial c_{i}}=0.$$

Then the unknown parameters can be obtained by the LSR estimators.

### 3.3. Performance Analysis

**Figure 2:**
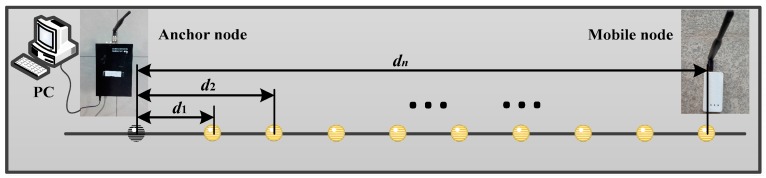
Experimental platform for the measured distance of WSN.

In order to evaluate the measured distance error model, the measured characteristic of chips for detection wireless signal is configured to eliminate the measurement deviations. The accurate distance values between the mobile and anchor nodes are calculated for the implementation of TOA solution methods. Note that one of the anchor nodes is attached to a support structure fixedly and the mobile node moves every 0.6 m; we can read the direct distance between the anchor node and the mobile node through data acquisition software on a computer. Moreover, the mobile node moves along a straight line, and the maximum distance of movement is 18.6 m. The WSN experimental platform for the measured distance is depicted in Figure 2. At every measurement point, we always perform 20 measurements and take an average value.

**Figure 3:**
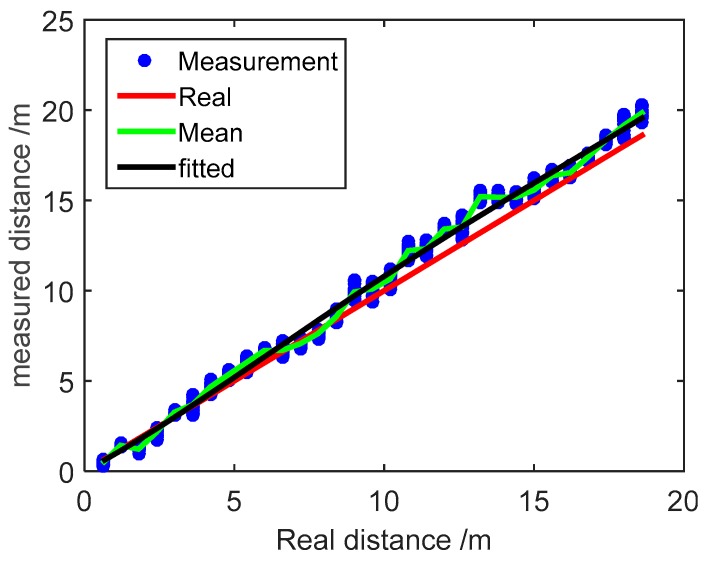
Measured distance values with WSN.

The measured distance values between the anchor node and mobile node are plotted in Figure 3. The measured values of every sampling point are represented by a series of blue dots, then the average in every measurement point is expressed by a green line. From Figure 3, we observe that all measured distance values are situated around the corresponding real values, which are expressed as a red line. Note that in this case, the average error of measured distance is 0.66 m, compared with the actual values. The polynomial fitting method with quartic polynomial spline curve is applied to smooth the measured distance value, and the fitted result is expressed as a black line. The curve fitted by the quartic polynomial spline shows increasing systematic error as the measured distance increases, in comparison with the actual value. The average error of the fitted results is 0.60 m. As the mobile node moves away from the anchor node, the measured distance errors show a continuous increase due to the fact that the wireless signal can suffer from reflection, obstacles, and multipath propagation. 

**Figure 4:**
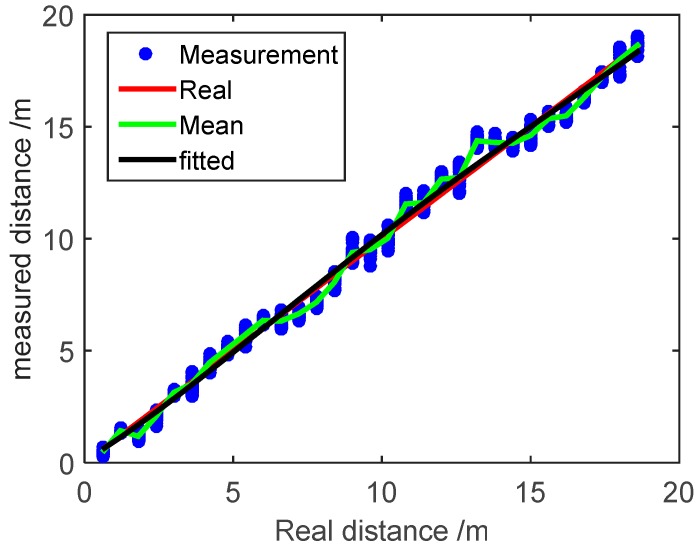
Measured distance values after the LSR correction.

According to the measured distance model described in Section 3.1, the systematic error of measured distance values can be corrected based on the least squares regression (LSR), then the measurement error parameters (*f*, *c_i_*) proposed in Section 3.2 can be obtained. The measured distance values after the LSR correction are shown as Figure 4. After the LSR correction, the measured distance values between the anchor node and mobile node are located more closely around the real value and the average error is decreased to 0.18 m. The curve fitted by the quartic polynomial spline nearly covers the real distance curve. As a consequence, the systematic error of measured distance values can be estimated by the LSR algorithm.

## 4. Fuzzy Adaptive Tightly-Coupled Integrated Model

### 4.1. Structure of Tightly-Coupled Integration

**Figure 5:**
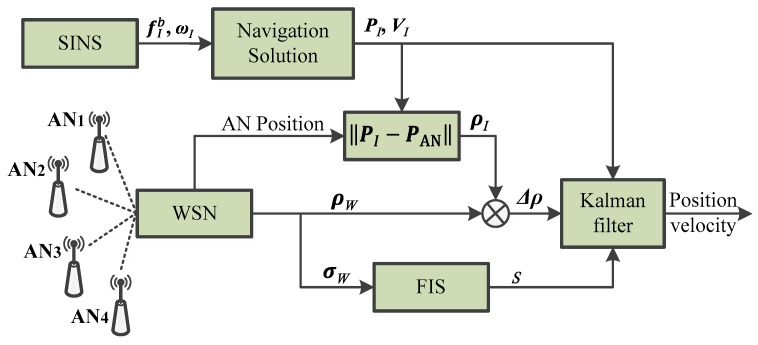
Illustration of the SINS/WSN FATCI system.

The SINS/WSN FATCI architecture is shown in Figure 5. Firstly, the position and velocity of SINS can be calculated through the navigation solution algorithm, which uses the specific force (denoted as $f_{I}^{b}$) and angular rate (denoted as ***ω****_I_*). Meanwhile, the measured distances (denoted as ***ρ****_W_*) between the four anchor nodes and the mobile node are obtained by WSN. Moreover, the covariance of ***ρ****_W_* can also be calculated. Secondly, the pseudo-distances (denoted as ***ρ****_I_*) between the SINS and four anchor nodes are calculated based on the positions of anchor nodes and the position of a mobile target measured by SINS. The difference (**Δ*ρ***) between the pseudo-distance (***ρ****_I_*) and measured distance (***ρ****_W_*) is the observation value of the Kalman filter. Obviously, the differences (**Δ*ρ***) of two distances between the mobile node and four anchor nodes are mutually independent and not relevant. Finally, the fuzzy inference system (FIS) controls the coefficient (denoted as *s*) to adjust the observation covariance matrix of the Kalman filter, according to the statistical covariance of measured distance for WSN. Hence, the adaptive tightly-coupled integration model can be obtained through the fuzzy adaptive Kalman filter (FAKF).

### 4.2. State-Space Model

According to the WSN measured distance model between the anchor nodes and mobile node, we assume that the discrete NLOS error transfer equation for the measured distance model is represented as

(17) $$\varepsilon_{d}\left(k+1\right)=\varepsilon_{d}\left(k\right)+\nabla_{\varepsilon }\left(k\right),$$

where the $\nabla_{\varepsilon }\left(k\right)$ is the Gaussian white noise.

Consequently, the state equation of tightly-coupled integrated system is obtained based on Equations (4) and (17) and is expressed as

(18) $$\underset{x^{t}\left(k+1\right)}{\underbrace{[\begin{array}{c} x\left(k+1\right)\\ \varepsilon_{d}\left(k+1\right) \end{array}]=}}\underset{F_{k,k+1}^{t}}{\underbrace{\left[\begin{array}{cc} F_{k,k+1} & 0\\ 0 & 1 \end{array}\right]}}\underset{x^{t}\left(k\right)}{\underbrace{\left[\begin{array}{c} x\left(k\right)\\ \varepsilon_{d}\left(k\right) \end{array}\right]}}+\underset{G_{k}^{t}}{\underbrace{\left[\begin{array}{cc} G_{k} & 0\\ 0 & 1 \end{array}\right]}}\underset{W^{t}\left(k\right)}{\underbrace{\left[\begin{array}{c} W_{k}\\ \nabla_{\varepsilon }\left(k\right) \end{array}\right]}},$$

where $W^{t}\left(k\right)$ is the Gaussian white noise with zero mean, and its covariance matrix is Q(k). The mark *t* is represented as the tightly-coupled integration.

Because of the SINS/WSN integrated positioning system, the position of the mobile node measured by SINS is assumed to be $\left(x_{I},y_{I},z_{I}\right)$. Meanwhile, the position of *i*-th anchor node is set to $\left(x_{i},y_{i},z_{i}\right)$. Mathematically, the pseudo-distance (denoted as $\rho_{Ii}$) betwee then *i*-th anchor node and the mobile node is expressed as

(19) $$\rho_{Ii}={\left({\left(x_{I}-x_{i}\right)}^{2}+{\left(y_{I}-y_{i}\right)}^{2}+{\left(z_{I}-z_{i}\right)}^{2}\right)}^{1/2}.$$

Then, Equation (19) is used for the first-order Taylor expansion at the point $\left(x_{I},y_{I},z_{I}\right)$ [22]. Namely,

(20) $$\rho_{Ii}\approx {\tilde{\rho }}_{Ii}={\left({\left(x_{I}-x_{i}\right)}^{2}+{\left(y_{I}-y_{i}\right)}^{2}+{\left(z_{I}-z_{i}\right)}^{2}\right)}^{1/2}+\frac{\partial \rho_{Ii}}{\partial x}\delta x+\frac{\partial \rho_{Ii}}{\partial y}\delta y+\frac{\partial \rho_{Ii}}{\partial z}\delta z,$$

where ${\tilde{\rho }}_{Ii}$ is the approximate value of ${\tilde{\rho }}_{Ii}$. Because δx, δy, and δz are very small and close to zero, we can conclude that ${\tilde{\rho }}_{Ii}$ is equal to $\rho_{Ii}$. According to the measured distance model of WSN described in Equation (13), the distance between *i*-th anchor node and mobile node measured by WSN is

(21) $$\rho_{Wi}=d_{i}+\varepsilon_{d}+\upsilon_{d},$$

where $d_{i}$ is the real distance from mobile node to *i*-th anchor node; $\varepsilon_{d}$ is the NLOS error; $\upsilon_{d}$ is the measured noise; and $\upsilon_{d}=c_{i}+\Delta_{i}$. Hence, let us define the $\Delta \rho_{i}$ as

(22) $$\Delta \rho_{i}=\rho_{Ii}-\rho_{Wi}=\frac{\partial \rho_{Ii}}{\partial x}\delta x+\frac{\partial \rho_{Ii}}{\partial y}\delta y+\frac{\partial \rho_{Ii}}{\partial z}\delta z-\varepsilon_{d}-\upsilon_{d},$$

where

(23) $$\frac{\partial \rho_{Ii}}{\partial x}=\frac{x-x_{i}}{\rho_{Ii}},\;\frac{\partial \rho_{Ii}}{\partial y}=\frac{y-y_{i}}{\rho_{Ii}},\;\frac{\partial \rho_{Ii}}{\partial z}=\frac{z-z_{i}}{\rho_{Ii}}.$$

The measurement equation of tightly-coupled integrated positioning system can be expressed as

(24) $$z^{t}\left(k\right)=H_{k}^{t}x^{t}\left(k\right)+V^{t}\left(k\right),$$

where $z^{t}\left(k\right)={\left[\begin{array}{ccc} \begin{array}{cc} \Delta \rho_{1} & \Delta \rho_{2} \end{array} & \ldots & \Delta \rho_{n} \end{array}\right]}^{T}$,

$$V^{t}\left(k\right)={\left[\begin{array}{ccc} \begin{array}{cc} \upsilon_{d1} & \upsilon_{d2} \end{array} & \ldots & \upsilon_{dn} \end{array}\right]}^{T},$$

$$H_{k}^{t}=\left[\begin{array}{ccc} \begin{array}{ccc} \partial \rho_{I1}/\partial x & \partial \rho_{I1}/\partial y & \partial \rho_{I1}/\partial z\\ \vdots & \vdots & \vdots \\ \partial \rho_{In}/\partial x & \partial \rho_{In}/\partial y & \partial \rho_{In}/\partial z \end{array} & 0_{n\times 6} & \begin{array}{c} -1\\ \vdots \\ -1 \end{array} \end{array}\right].$$

### 4.3. FAKF Algorithm

Note that a state-space equation is combined with a system-state model and a measurement model; the equations of the system model are constructed as follows:

(25) $$\{\begin{array}{c} x\left(k+1\right)=F_{k,k+1}x\left(k\right)+G_{k}W\left(k\right)\\ z\left(k\right)=H_{k}x\left(k\right)+V\left(k\right) \end{array}$$

Here, it is obvious that the state-space equation is a linear system. The KF is one of the most common filtering methods for linear systems; equations of KF constitute two groups: time update equations and measurement update equations [23]. The time update equations are summarized as follows:

(26) $${\hat{x}}_{k|k-1}=F_{k,k-1}{\hat{x}}_{k-1|k-1}$$

(27) $$P_{k|k-1}=F_{k,k-1}P_{k-1|k-1}F_{k,k-1}^{T}+G_{k}Q_{k-1}G_{k}^{T},$$

where ***Q****_k_*_−1_ is a covariance matrix of process noise and ***P****_k|k_*_−1_ is an a posteriori error covariance matrix (a measure of the estimated accuracy of the state estimate).

The measurement update equations are written as

(28) $${\hat{x}}_{k|k}={\hat{x}}_{k|k-1}+K_{k}\left[z_{k}-H_{k}{\hat{x}}_{k|k-1}\right]$$

(29) $$P_{k|k}=\left[I-K_{k}H_{k}\right]P_{k|k-1}$$

(30) $$K_{k}=P_{k|k-1}H_{k}^{T}\left[H_{k}P_{k|k-1}H_{k}^{T}+R_{k}\right],$$

where ***K****_k_* is an optimal Kalman gain and ***R****_k_* is a covariance of the observation noise ***V***, which is assumed to be zero mean Gaussian white noise.

**Figure 6:**
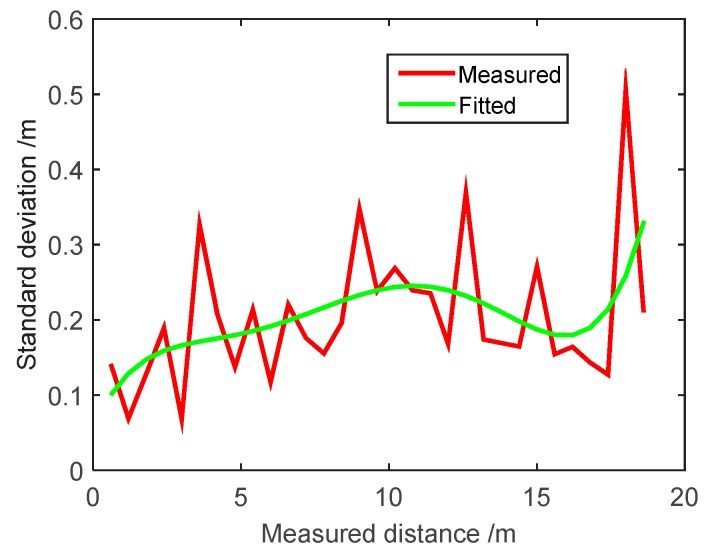
Standard deviation of measured distance for WSN.

According to the well-known Kalman filter algorithm, we can observe that the measurement covariance error matrix ***R****_k_* is able to decide the confidence level of measurement in the process of estimation. For the SINS/WSN tightly-coupled integrated system, the covariance of measured distance errors between the anchor nodes and the mobile node can influence positioning accuracy by means of the confidence level of measured distance. The standard deviation of WSN measured distance value is shown in Figure 6. The standard deviation of measured distance value is calculated based on measurements, which are performed 20 times at every measurement point, and represented as a red line. The measured process is the same as that described in Section 3.3. The polynomial fitting method with a five-degree polynomial spline curve is applied to smooth the standard deviation value, and the fitted result is expressed as a green line. The curve fitted by the five-degree polynomial spline experiences a continuous change as the measured distance increases. As a consequence, the changed continuous standard deviation is configured to take advantage of the different confidence levels for different measured distance values.

In order to improve the positioning accuracy for the SINS/WSN tightly-coupled integrated system, the fuzzy adaptive kalman filter (FAKF) method is built with a fuzzy inference system (FIS) based on the statistics covariance of WSN measured distance. The FIS is used to control the measurement covariance error matrix ***R****_k_* to change the confidence level for the measured distance. If one of the measured distances performs a lower covariance, the corresponding parameter of ***R****_k_* will become smaller to get a higher confidence level for this measurement. FIS is employed to adjust the measurement covariance error matrix ***R****_k_*. The input of FIS is the fitted standard deviation of the measured distance value (denoted as $\sigma_{W,k}$). The FIS is a single-input and single-output (SISO) structure and the output of FIS is coefficient *S_k_*. The fuzzy rules of coefficient *S_k_* can be defined as:

If $\sigma_{W,k}$ is EQ, then *S_k_* is EQ;
If $\sigma_{W,k}$ is MO, then *S_k_* is MO;
If $\sigma_{W,k}$ is LE, then *S_k_* is LE.

**Figure 7:**
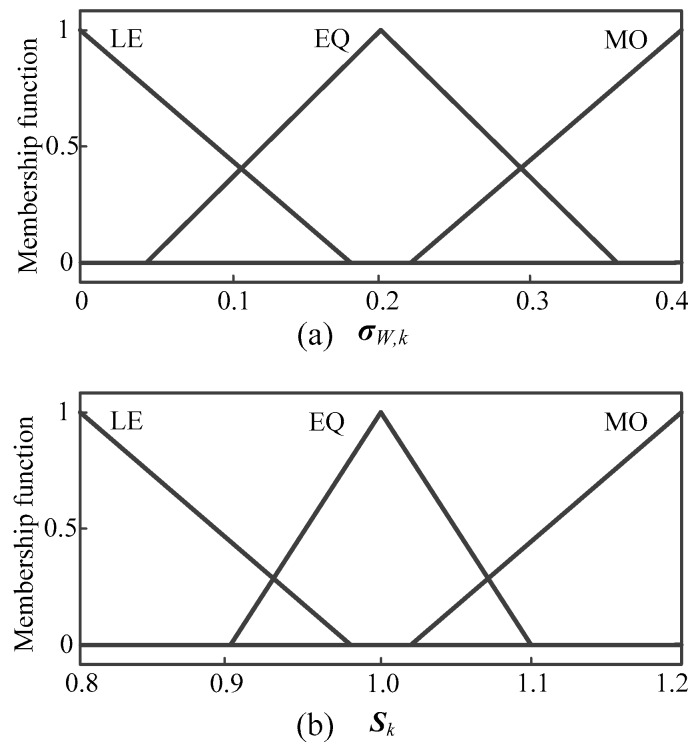
Membership functions of input and output variable of FIS: (**a**) Membership functions of input $\sigma_{W,k}$; (**b**) membership functions of output *S_k_*.

**Figure 8:**
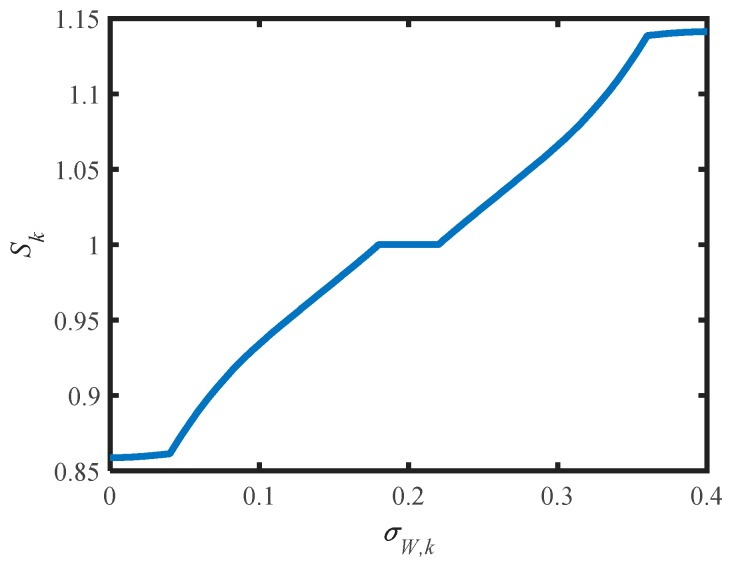
Control rule of FIS. $\sigma_{W,k}$ is the input of FIS; *S_k_* is the output of FIS.

In Figure 7a,b, the membership functions of $\sigma_{W,k}$ and *S_k_* are the triangles. We choose the gravity method to calculate the ambiguity resolution, which means that the output value is the gravitational center of the area that is enclosed by membership function curve. Finally the control rule of FIS can be calculated through fuzzification, fuzzy inference, and defuzzification. Then the control rule of FIS can be shown as in Figure 8. Obtained from the FIS operation each time, *S_k_* is put into Equation (31) to adjust ***R****_k_* adaptively:

(31) $$R_{k+1}=S_{k+1}^{b}R_{k},$$

where coefficient *b* is used to control the degree of amplification. Note that the change rate of observation noise matrix is transformed by the value *b* which is set up artificially. According to the FAKF filter method of the WSN/SINS tightly-coupled integrated positioning system, we summarize the developed filtering algorithm in Algorithm 1.
**Algorithm 1.** Summary of the SINS/WSN FATCI algorithm.1: Initialize state vector
${\hat{x}}_{0}$ and covariance matrix ***P***_0_2: **for**
*k* = 1, 2, 3, … **do**3:   Obtain
$\hat{z}$ and $\hat{\sigma }$ from WSN measurement4:   **if**
$\hat{z}\neq z_{k-1}$
**then**5:     Evaluate
$\hat{z}$ and get the number of measurement6:        
$z_{k}=\hat{z}$, $\sigma_{W,k}=\hat{\sigma }$
7:     Calculate
$S_{k}$ from FIS based on the $\hat{\sigma }$8:     Update the measurement covariance error matrix          $R_{k+1}=S_{k+1}^{b}R_{k}$9:     Compute Kalman gain matrix       $K_{k}=P_{k|k-1}H_{k}^{T}\left[H_{k}P_{k|k-1}H_{k}^{T}+R_{k}\right]$10:      Update measurement equations using (28) and (29)11:      Predict state equations using (26) and (27)12:   **end if**13: **end for**

## 5. Experiments

In this section, the performance of proposed tightly-coupled integrated positioning system was evaluated. Firstly, the experimental platform for the SINS/WSN integrated positioning system was built using MEMS-based IMU and WSN with chirp spread spectrum (CSS) technology. The MEMS-based IMU have the advantages of small size, data stabilization, and low cost compared with other IMUs for mobile target localization in the enclosed environment. CSS signal of WSN can be used for the localization in indoor facilities and coal mine with some advantages, such as high temporal resolution, anti-multipath capability, high data rate, low power, and so on. Furthermore, some tests for the SINS/WSN experimental platform can be implemented based on the loosely-coupled integration and tightly-coupled integration, as well as the FATCI models.

### 5.1. Experimental Setup

**Figure 9:**
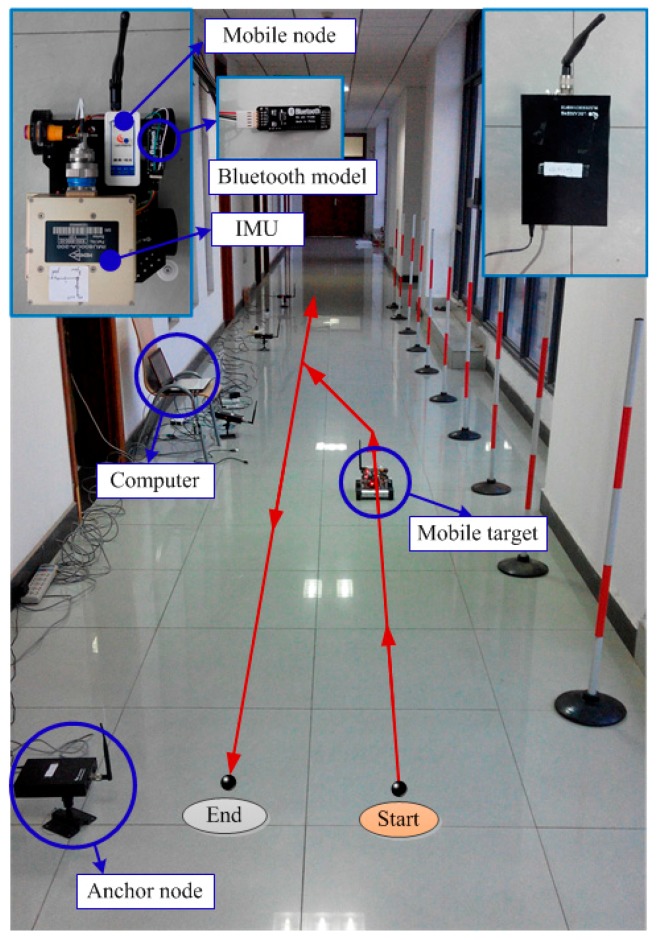
Experimental diagram of integrated positioning system with SINS/WSN.

In order to implement the experiments, an electric vehicle has been used as the mobile target. The initial parameters of the positioning system were given as:

(1) The WSN consisted of four anchor nodes and a mobile node. The mobile node was placed on the mobile vehicle and the anchor nodes were deployed along the corridor. A long and narrow location area held four anchor nodes. Time synchronization for TOA approach among the anchor nodes can be accomplished through the Ethernet cable connection. The power was supplied for the anchor nodes through the twisted pair and the mobile node was operated from batteries. The sampling period of WSN was 0.1 s.
(2) The chosen SINS is a six-degrees-of-freedom (6DoF) Inertial Measurement Unit providing accurate monitoring of angular rate and linear acceleration in any orientation. The IMU incorporates advanced MEMS rate gyro technology resulting in exceptional reliability and performance, with in-run bias stability of <3°/h. Typical applications include platform stabilization, dynamic testing, and avionics. The acceleration resolution is less than 1/2000 of the absolute value of the gravitational acceleration. The RS232 serial communication was used only for data transmission between IMU and computer. The baud rate was 115,200 bit/s, and the sampling period was 0.01 s. The power was supplied for the SINS through a storage battery in the electric vehicle.
(3) The IMU was installed on the mobile vehicle and the inertial data was transported with two Bluetooth models. One Bluetooth model was connected to the IMU with RS232 ribbon cable, the other was connected to the computer by a wired USB–serial connection (Bluetooth 1.1 and USB 2.0). The maximum received distance between two Bluetooth models is up to 60 m in ideal conditions (free space). The wireless signal was broadcasted by the mobile node in real time and received by the anchor nodes. The distance values between the mobile node and anchor nodes were firstly collected by anchor nodes and then forwarded to the switch, which was connected to a computer via an Ethernet cable. Additionally, the inertial data of IMU and the distance value of WSN were used to calculate the fusion center, which was a computer. As a consequence, the position and attitude of the mobile vehicle were obtained by means of the integration algorithm. Figure 9 shows an experimental diagram of an integrated positioning system with SINS/WSN.

The starting point of a mobile vehicle was set as (0, 0) m. The trajectory of the mobile vehicle is shown as the red line in Figure 9. The experiment lasted 60 s.

### 5.2. LSR Correction Model for WSN Measured Distance

Note that the distance measurement accuracy of WSN was able to influence the positioning accuracy for the SINS/WSN integrated system. According to the error correction model for measured distance that is proposed in Section 3.2, the LSR correction model for WSN can be used to evaluate positioning performance based on the WSN-only experimental platform.

**Figure 10:**
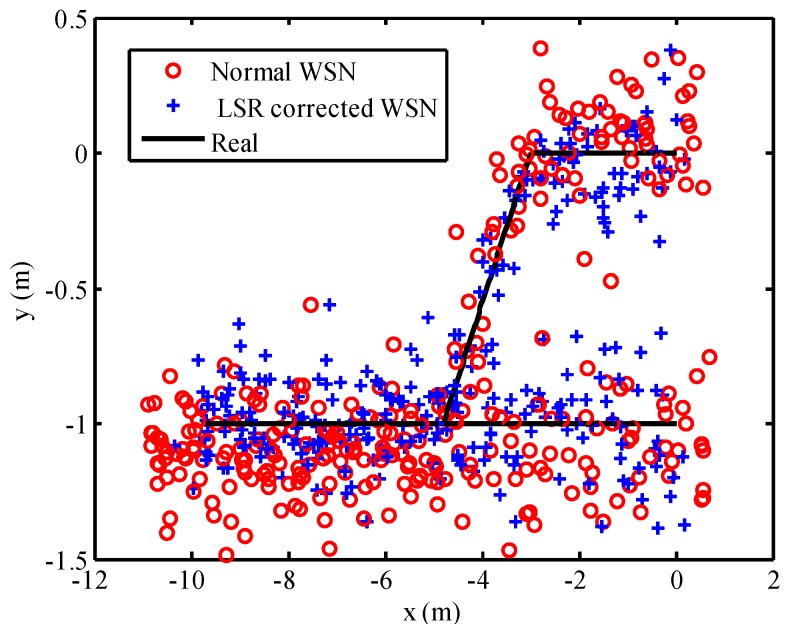
Performance of WSN with LSR correction model.

**Figure 11:**
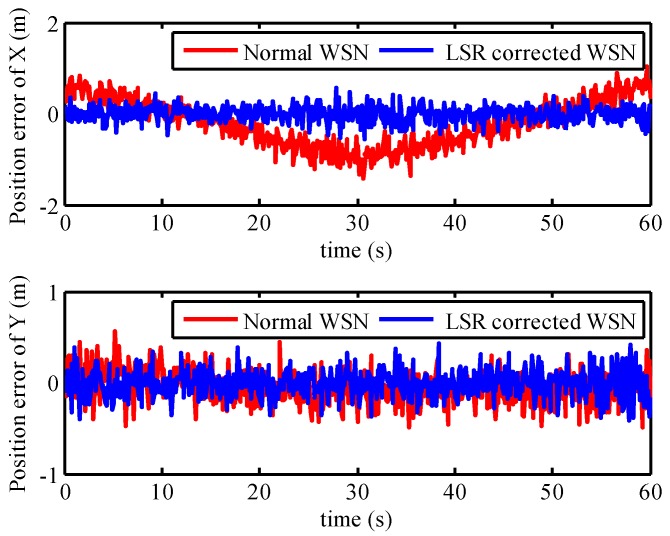
Position error of WSN with LSR correction model.

Anchor nodes received the wireless signal from the mobile node that was installed on the mobile vehicle. The TOA-based measured distance value can be calculated by a programmable control unit in the anchor node. Using the LSR correction model for the measured distance, the measurement error of anchor nodes was corrected and the positioning results of WSN are presented in Figure 10. Red circles represented the positioning result of uncorrected WSN, which applied to the original measured distance readings. Meanwhile, the positioning result corrected by the LSR correction model was represented as blue crosses. There are a large number of red circles defecting from the general trajectory in Figure 10. Furthermore, the red circles deviated from the black line more seriously than the blue crosses near the point (−10, −1) m. The position errors of WSN with the uncorrected model and LSR correction model are plotted in Figure 11. From this figure, it is obvious that the position errors with uncorrected WSN were composed of the systematic error and stochastic error, where the systematic error changed over time. However, the position error of WSN updated by the LSR has little systematic error. The maximum position error and its variance with uncorrected WSN were 1.26 m and 0.2618, respectively. On the contrary, the maximum position error and its variance with LSR correction algorithm were 0.55 m and 0.0261, respectively. Note that, in this case, the positioning accuracy of WSN with LSR correction model is much higher than that with uncorrected WSN, after we took into account the position error and its variance. This explains why the LSR correction model can decrease the positioning error of WSN.

### 5.3. Loosely-Coupled Integration Result

**Figure 12:**
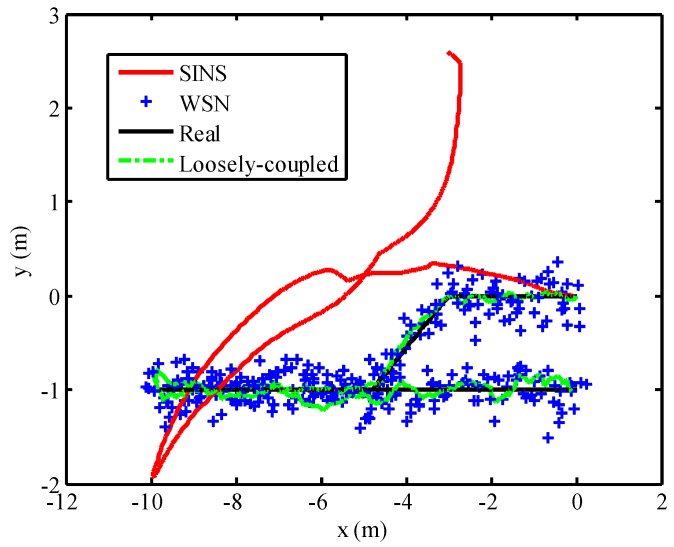
Positioning performance using SINS/WSN loosely-coupled integration model.

**Figure 13:**
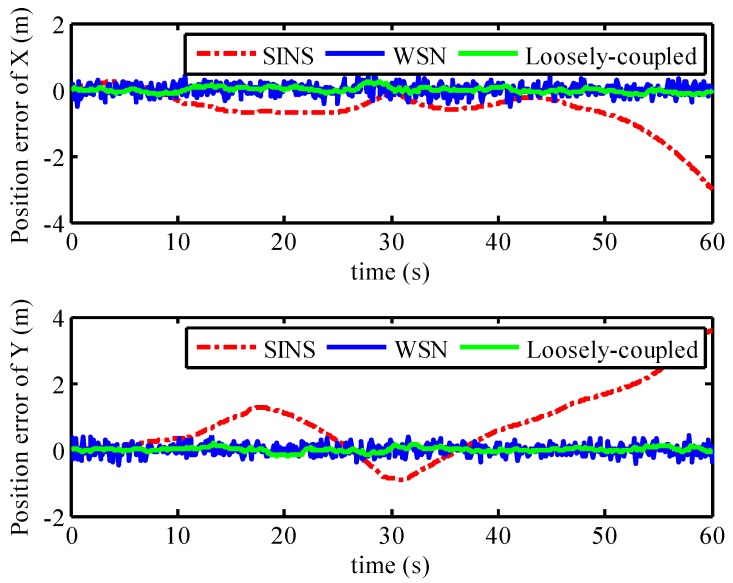
Position error of the SINS/WSN loosely-coupled integration model.

Combined with the positioning result of WSN with LSR correction model, the loosely-coupled integrated model was built based on the position and attitude of SINS. The loosely-coupled integrated model has been described in Section 2. In order to analyze the positioning performance of the loosely-coupled integrated model with SINS/WSN system, the fusion result of loosely-coupled integrated model is depicted in Figure 12, combined with the positions using SINS-only and WSN-only. The positioning result of WSN with the LSR correction model was represented as a series of blue crosses. The result of pure SINS, expressed as a red line, already experienced drifted position error after the mobile target started to move. The position error of SINS became seriously divergent as time went by; however, the position result of WSN experienced stochastic error but not drifted error. The positioning result of the loosely-coupled integration model is expressed as a green line and can track the real trajectory effectively with small position error. The position error of the SINS/WSN loosely-coupled integrated model is shown in Figure 13. Obviously, the positioning accuracy of the loosely-coupled integrated positioning system was better than that of the SINS and WSN.

### 5.4. FATCI Model with WSN Short-Term Failure

Note that the SINS/WSN loosely-coupled integrated positioning system has the better tracking accuracy, compared with the single operation of SINS and WSN. However, the loosely-coupled integrated model that observed the final position of WSN will lead to the failure of the integrated model when the WSN cannot calculate the position of a mobile target. Because of the failure or sheltered barrier for the anchor node, the number of anchor nodes that can receive the wireless signal was less than four on a two-dimensional plane. In order to evaluate the positioning performance with loosely-coupled integration and tightly-coupled integration, as well as the FATCI algorithms for WSN short-term failure, we interrupted the measured distance value of second anchor node in the process of motion. The failure time of the second anchor node was set from 18 s to 23 s, as well as from 40 s to 45 s. Then the final position of WSN cannot be obtained during these two time segments except for the measured distance values from other anchor nodes. The traditional tightly-coupled positioning model cannot adjust the measurement covariance error matrix based on the measuring accuracy of every anchor node.

**Figure 14:**
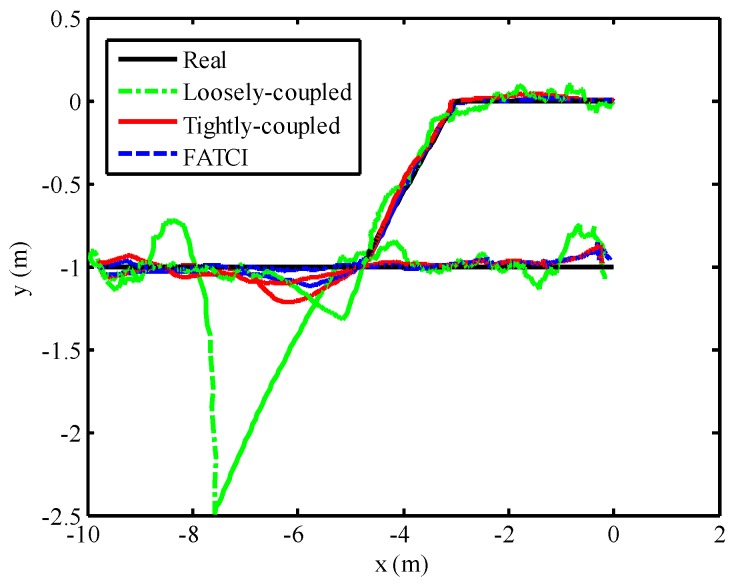
Positioning performance with different integrated methods based on WSN short-term failure.

**Figure 15:**
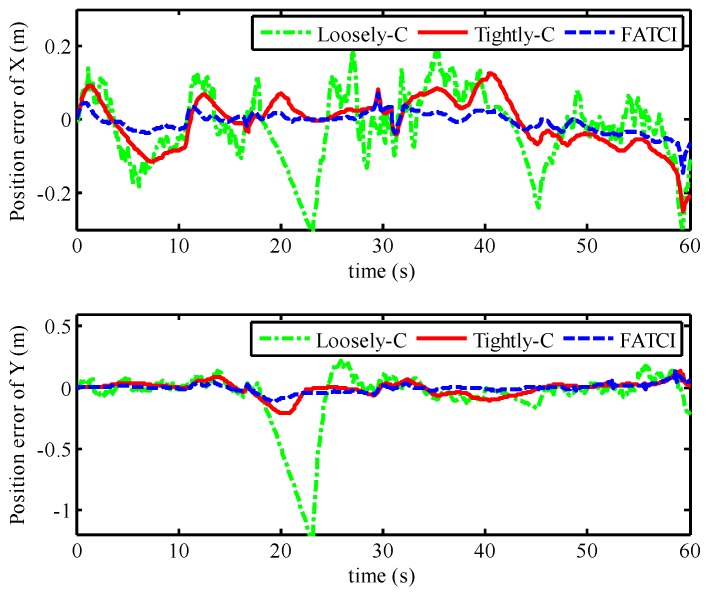
Position error with different integrated methods based on WSN short-term failure.

The positioning performance of the SINS/WSN integrated system with different integrated methods including loosely-coupled, tightly-coupled, and FATCI is shown in Figure 14. From this figure, we can see that the positioning result with the loosely-coupled method has expressed the large divergence at the failure time of WSN, shown as a green line. Nevertheless, that of the loosely-coupled method can track the real trajectory besides the failure time. The positioning results of tightly-coupled and FATCI methods can follow the real trajectory effectively without the drifted error. The position errors of SINS/WSN integrated system with different integrated methods are shown in Figure 15. The maximum position error with loosely-coupled integration model was situated at 1.49 m, because of the drifted position error. The maximum position errors with tightly-coupled integration and FATCI model were 0.25 m and 0.15 m, respectively. Due to the fact that the FATIC model applied both FIS and statistic covariance of measured distance value to adjust the measurement covariance error matrix ***R****_k_* of the Kalman filter, the influence from different noises of measured distance would be weakened and the confidence level for every measurement would be changed based on the measured accuracy. The FATCI method reduces the position error by about 40% compared with the traditional tightly-coupled integration method.

### 5.5. FATCI Model with WSN Normal Condition

**Figure 16:**
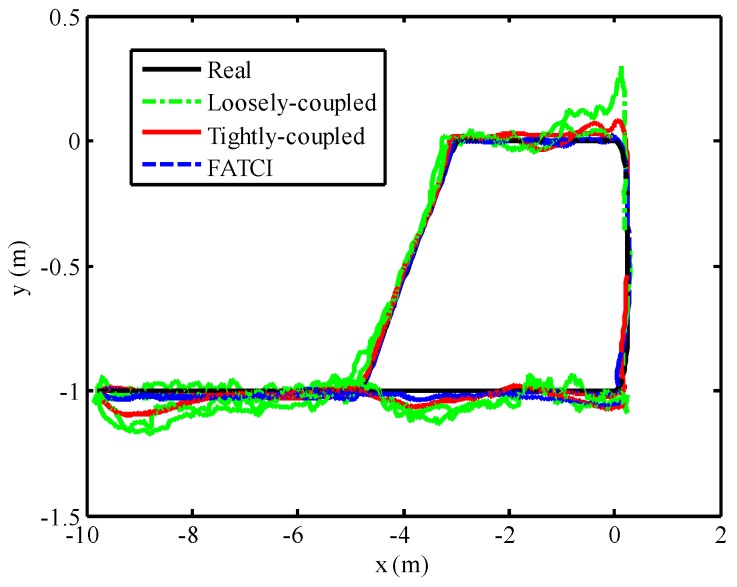
Positioning performance with different integrated methods based on WSN normal conditions.

With the aim of analyzing the positioning performance of the FATCI model more effectively, we have operated the SINS/WSN experimental platform with WSN normal condition. In order to illustrate the experimental performance of integrated positioning system more effectively, the duration time of the experiment has been increased to 127 s. The mobile target moved along with the pre-set trajectory for two loops. In the course of the experiment, the influence of WSN from reflection and the multipath of the wireless signal were considered adequate. Meanwhile, every anchor node can receive the wireless signal broadcasted by the mobile node in the whole process of motion. So the loosely-coupled integration model can be achieved all the time. The performance of SINS/WSN integrated positioning system with loosely-coupled, tightly-coupled, and FATCI models is shown in Figure 16. The positioning results with loosely-coupled, tightly-coupled, and FATCI models are represented by a green dashed line, a red line, and a broken blue line, respectively. It is obvious that all the positioning results with different integration methods can track the real trajectory, which is represented by a black line. Note that the positioning trajectory of loosely-coupled and tightly-coupled methods produced larger position error at some corners away from the real trajectory.

**Figure 17:**
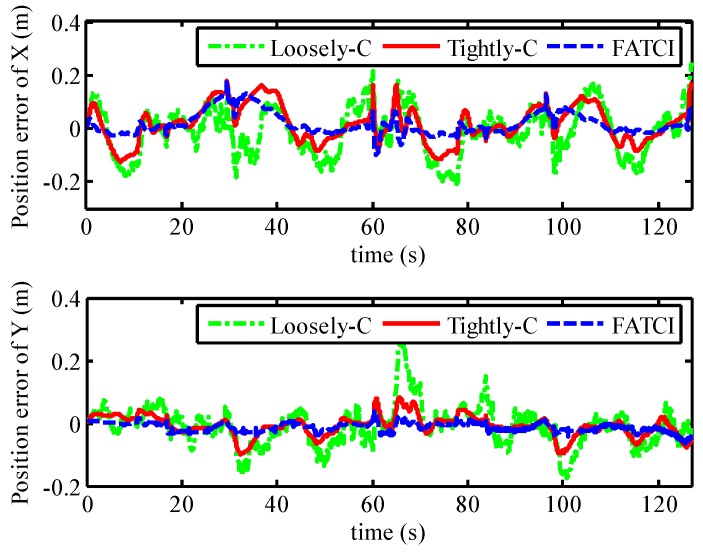
Position error with different integrated methods based on WSN normal conditions.

**Figure 18:**
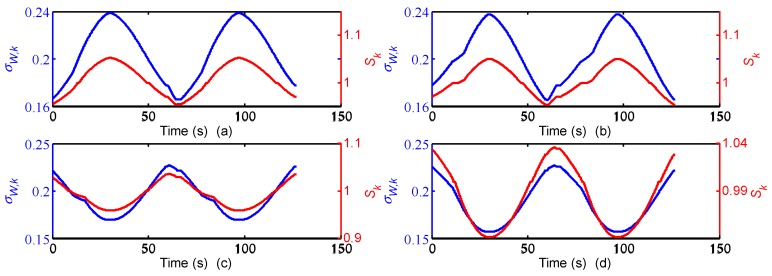
Input and output result of FIS, (**a**) first anchor node; (**b**) second anchor node; (**c**) third anchor node; (**d**) fourth anchor node.

The position errors for the SINS/WSN integrated system were calculated with different methods and plotted in Figure 17. From this figure, the positioning system based on the FATCI model can track the real trajectory with smaller position error. On the contrary, the loosely-coupled and tightly-coupled models have produced some serious errors in the process of motion. We can observe that the position error with the FATCI model was less than that with loosely-coupled and tightly-coupled models because the FATCI model can adjust the measurement covariance error matrix ***R****_k_* of the Kalman filter based on the accuracy of distance measured in WSN. The results for input *σ_W,k_* and output *S_k_* of the FIS are shown in Figure 18. The parameter *S_k_* of every anchor node changed along with the covariance *σ_W,k_* of measured distances, according to the control algorithm of FIS. Meanwhile, the change rules for the parameter *S_k_* of every anchor node were not the same but depended on the measured distance from the mobile node to every anchor node. The measurement covariance error matrix ***R****_k_* of the Kalman filter and a confidence level of measurements can be adjusted adaptively. As a result, the positioning accuracy of the FATCI model increased. 

The maximum position errors with the loosely-coupled, tightly-coupled, and FATCI models were 0.3748 m, 0.2344 m, and 0.1488 m, respectively. Note that, in this case, the accuracy of the positioning system with the FATCI model obviously surpassed that with loosely-coupled and tightly-coupled integration models, with 60.3% and 36.5% improvements, respectively. Table 1 shows a performance comparison for different integration models based on the SINS/WSN integrated positioning system.

## 6. Conclusions

This paper has proposed a FATCI positioning system using SINS/WSN, aimed at rectifying the low stability of a loosely-coupled model and the low accuracy of the traditional tightly-coupled model. The main contribution of the paper is summarized as follows: Firstly, the measured distance correction model of LSR was built based on a series of WSN tests for range measurement. The systematic error of measured distance has been corrected and the positioning accuracy of pure WSN increased. Secondly, the tightly-coupled integration model of SINS/WSN was established with the corrected measured distance between every anchor node and the mobile node. Then the stability of the integrated positioning system was enhanced for the WSN short-term failure. Finally, the FIS was utilized to adjust the confidence level of the Kalman filter for WSN range measurement. As a consequence, the FATCI model for SINS/WSN was finished. 

We have evaluated the performance of the proposed FATCI model using a mobile target in an indoor environment. The experimental results have shown that the loosely-coupled integrated positioning system can track the real trajectory and overcome the disadvantages of SINS-only and WSN-only. However, the loosely-coupled integration model produced drifted error for WSN short-term failure compared with the traditional tightly-coupled and proposed FATCI models. For the normal WSN, the maximum position errors with the loosely-coupled, traditional tightly-coupled, and FATCI models were 0.3748 m, 0.2344 m, and 0.1488 m, respectively. The accuracy of the positioning system with the FATCI model obviously surpasses that of the loosely-coupled and traditional tightly-coupled integration models, with 60.3% and 36.5% improvements, respectively.

## Figures and Tables

**Table 1:** Performance comparison for different integration models.

Item		Loosely-Coupled	Tightly-Coupled	FATCI
**Position error range (m)**	***x***	−0.2324~0.2499	−0.1224~0.1870	−0.0986~0.1756
	***y***	−0.1816~0.3396	−0.0980~0.0873	−0.0641~0.0384
**Variance**	***x***	0.0077	0.0049	0.0017
	***y***	0.0052	0.0011	0.0002
**Maximum (m)**		0.3748	0.2344	0.1488
**Average (m)**		0.0956	0.0682	0.0384
